# COVID-19 Interference with Renin-Angiotensin System in the Context of Heart Failure

**DOI:** 10.1128/mBio.00946-20

**Published:** 2020-05-22

**Authors:** Eftychios Siniorakis, Spyridon Arvanitakis, Ioannis Nikolopoulos, Maximilianos Elkouris

**Affiliations:** a Department of Cardiology, Sotiria Chest Diseases Hospital, Athens, Greece; b Second Department of Infectious Diseases, Sotiria Chest Diseases Hospital, Athens, Greece; c Department of Clinical Biochemistry, Sotiria Chest Diseases Hospital, Athens, Greece; Johns Hopkins Bloomberg School of Public Health

## LETTER

Novel coronavirus severe acute respiratory syndrome coronavirus 2 (SARS-CoV-2) (coronavirus disease 2019 [COVID-19] pandemic) attacks the host cells modulating the local renin-angiotensin (RAS) system. Pending a definitive antivirus therapy, Fedson and colleagues propose in their recent article (1) a combination of generic drugs such as statins and angiotensin receptor blockers (ARBs) to prevent catastrophic cellular damage. The proposal could be applicable in a model of heart failure (HF) patients infected by coronavirus. These patients belong to the most vulnerable groups while concentrating in their RAS all the parameters sustained by Fedson and colleagues. First, most HF patients are treated with angiotensin-converting enzyme (ACE) inhibitors (ACEis) or ARBs, recently enriched with the novel compound sacubitril to form the prototype of ARBs/neprilysin (NEP) inhibitors (ARNIs) (2). Furthermore, a minority of HF patients receive statins which, however, are not an established treatment despite their immunomodulatory properties (3). All the aforementioned drugs aim at preventing the formation of deleterious angiotensins by deviating local RAS to the formation of innocuous products. To achieve this, two RAS components are needed, namely, ACE2 and NEP (4, 5). Both these proteases cleave angiotensin I and II into angiotensin (1–7) which is indispensable for cellular homeostasis (6). In fact, angiotensin (1–7) exerts antioxidant, anti-inflammatory, and antiproliferative effects, protecting target organs.

There are, however, some intriguing characteristics blemishing the role of ACE2 and NEP. ACE2 is an ACE homologue, unaffected by ACEis. Unexpectedly, in the case of the pandemic, ACEis become the main gate of coronavirus entry into the host cells. ACEis, ARBs, and statins all overexpress ACE2 on the surfaces of infected cells, increasing the virulence (7). Although most scientists discourage discontinuation of these treatments, a controversial debate is ongoing (8). However, this is not the end of the story. After its internalization, coronavirus forces ACE2 to downregulate in order to prevent further entries and guarantee controlled intracellular replications. This is when, according to Fedson’s theory, ARBs are called to sustain the survival of ACE2, maintaining a continuous production of angiotensin (1–7).

NEP has an equally complex role in the setting of HF patients infected by COVID-19. NEP catalyzes angiotensins to angiotensin (1–7) to a far greater extent than ACE2 (9). In the case of virus-induced ACE2 degeneration, NEP remains unaffected by the virus and is expected to undertake all the burden of angiotensin (1–7) production. This is questionable, nonetheless. Actually, 45 to 60% of HF patients are under treatment with ARNIs, a novel medication introduced in 2014, combining the ARB valsartan with sacubitril, a prototype NEP inhibitor (2, 10). Thus, in a scenario of ACE2 blockage by the virus and NEP inhibited by sacubitril, abrogation of angiotensin (1–7) will occur, with unpredictable consequences. We share with Fedson and colleagues their enthusiasm in proposing generic drugs in the setting of COVID-19 therapy. However, in the case of HF, therapeutic modulations of ACE2, NEP, and angiotensin (1–7) warrant further exploration.
